# Cardiovascular and Respiratory Toxicity of Protamine Sulfate in Zebrafish and Rodent Models

**DOI:** 10.3390/pharmaceutics13030359

**Published:** 2021-03-09

**Authors:** Joanna Miklosz, Bartlomiej Kalaska, Piotr Podlasz, Małgorzata Chmielewska-Krzesińska, Miłosz Zajączkowski, Adam Kosiński, Dariusz Pawlak, Andrzej Mogielnicki

**Affiliations:** 1 Department of Pharmacodynamics, Medical University of Bialystok, 15-089 Białystok, Poland; joanmiklosz@gmail.com (J.M.); bartlomiej.kalaska@umb.edu.pl (B.K.); dariuszpawlak@poczta.onet.pl (D.P.); 2 Department of Pathophysiology, Forensic Veterinary Medicine and Administration, Faculty of Veterinary Medicine, University of Warmia and Mazury in Olsztyn, 10-714 Olsztyn, Poland; piotr.podlasz@uwm.edu.pl (P.P.); malgorzata.chmielewska@uwm.edu.pl (M.C.-K.); 3 Department of Clinical Anatomy, Medical University of Gdansk, 80-210 Gdańsk, Poland; milosz.zajaczkowski@gumed.edu.pl (M.Z.); adam.kosinski@gumed.edu.pl (A.K.)

**Keywords:** cardiac toxicity, heparin, hERG, ion channels, protamine, respiratory toxicity, rodents, toxicity, zebrafish

## Abstract

Protamine sulfate (PS) is the only available option to reverse the anticoagulant activity of unfractionated heparin (UFH), however it can cause cardiovascular and respiratory complications. We explored the toxicity of PS and its complexes with UFH in zebrafish, rats, and mice. The involvement of nitric oxide (NO) in the above effects was investigated. Concentration–dependent lethality, morphological defects, and decrease in heart rate (HR) were observed in zebrafish larvae. PS affected HR, blood pressure, respiratory rate, peak exhaled CO_2_, and blood oxygen saturation in rats. We observed hypotension, increase of HR, perfusion of paw vessels, and enhanced respiratory disturbances with increases doses of PS. We found no effects of PS on human hERG channels or signs of heart damage in mice. The hypotension in rats and bradycardia in zebrafish were partially attenuated by the inhibitor of endothelial NO synthase. The disturbances in cardiovascular and respiratory parameters were reduced or delayed when PS was administered together with UFH. The cardiorespiratory toxicity of PS seems to be charge–dependent and involves enhanced release of NO. PS administered at appropriate doses and ratios with UFH should not cause permanent damage of heart tissue, although careful monitoring of cardiorespiratory parameters is necessary.

## 1. Introduction

Protamine sulfate (PS) is an alkaline–strong, arginine–rich, positively–charged protein isolated from salmon sperm. It is used to reverse the anticoagulant activity of unfractionated heparin (UFH) in case of threatening hemorrhage and to stabilize neutral protamine Hagedorn (NPH) insulin [1]. The chemical properties and animal origin of PS probably share responsibility for its adverse effects. In addition, PS binding to UFH can cause different reactions [2,3,4]. The most frequent clinical side effects of PS are systemic hypotension, decreased cardiac output, and bradycardia, however their mechanisms are still unclear [5]. The toxicity often manifests in diabetics, those allergic to fish, and vasectomized men, which may be associated with the immunogenicity of PS [6,7,8,9]. In these patients, the UFH neutralization may lead to rapid and life–threatening anaphylactic reactions [5,7]. Post–PS hemodynamic changes may result from direct histamine release, classic IgE–mediated anaphylaxis, or complement activation with secondary release of vasoactive substances [2,10,11,12,13]. The above reactions are probably also associated with respiratory toxicity, as manifested by pulmonary vasoconstriction leading to pulmonary hypertension and edema. This mechanism can be multifactorial and includes an increase of pulmonary endothelial cell permeability, release of vasoactive mediators such as cyclooxygenase and lipoxygenase metabolites, changes in the concentrations of calcium ions, or platelet accumulation in pulmonary circulation [2,14]. Some effects have been observed in vitro and in vivo, however the results are inconsistent [2,14,15]. This mismatch is due to different animal species and experimental models, dosage regimens, and time points of measurements used in various studies. Significant data were obtained from the patients undergoing cardiovascular procedures on the complex pharmacotherapy, which can mask the real effects of PS. Retrospective cohort studies or observations accompanying randomized clinical trials provide evidence for the incidence of toxicity, without explaining the direct cause or further implications. The mechanisms of post–PS reactions are still unknown and there are no guidelines for the management of these fatal consequences. Different preventive methods were used with less or more success, including skin tests, histamine blockers, steroids, indomethacin, methylene blue, and an initial low dose of PS exhausting the mediator [16,17,18,19,20]. Unfortunately none of these are widely accepted [17].

Due to its adverse effects, planning and implementation of prospective clinical trials with PS would be currently impossible and ethically unjustified. Possible responses of human organisms to administration of PS need to be recognized and appropriately managed. This encouraged us to explore in more detail potential cardiac, hemodynamic, and respiratory complications with the use of relevant in vitro and in vivo models. Our aim was to investigate the acute and chronic cardiorespiratory toxicity of PS in zebrafish, rats, and mice, as well as the possible mechanisms of its adverse effects.

## 2. Materials and Methods

### 2.1. Animals and Housing

Animals were obtained from the Center of Experimental Medicine in Medical University of Bialystok. They were bred in a 12 h light/dark cycle in a temperature– and humidity–controlled room, grouped cages as appropriate, and allowed to ad libitum access to sterilized tap water and standard chow in specific pathogen–free conditions. All procedures involving animals were approved by the local ethical committee (permit number: 2/2018) and conducted by Directive 2010/63/EU of the European Parliament and the Council on the Protection of Animals, ARRIVE (Animal Research: Reporting of In Vivo Experiments) guidelines, and the national laws. All animals were euthanized by exsanguination at the end of experiments.

### 2.2. Materials

We used protamine sulfate, N(ω)–nitro–l–arginine methyl ester, formaldehyde solution, hematoxylin solution (Sigma–Aldrich, Darmstadt, Germany), unfractionated heparin (UFH) (Polfa Warszawa, Warsaw, Poland), isoflurane (Baxter Polska, Warsaw, Poland), pentobarbital (Biovet, Pulawy, Poland), and phosphate-buffered saline (PBS) (Biomed Lublin, Lublin, Poland).

### 2.3. Zebrafish Experiment

**Figure 1:**
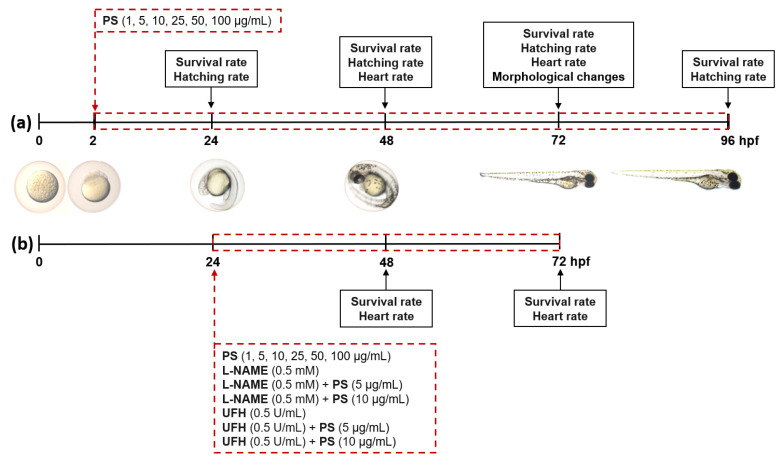
Schematic representation of 94 h (**a**) and 48 h (**b**) exposure of zebrafish to the full range of protamine sulfate (PS) concentrations, N(ω)–nitro–l–arginine methyl ester (l–NAME), and unfractionated heparin (UFH), alone or together with PS. Abbreviations: hpf, hour post-fertilization.

The wild–type zebrafish (Danio rerio) embryos of Tuebingen strain were exposed to PS from the 2nd to 96th hour post–fertilization (hpf; Figure 1a) or from the 24th to 72nd hpf (Figure 1b). PS was used at concentrations of 1, 5, 10, 25, 50, and 100 µg/mL; l–NAME at 0.5 mM; and UFH at 0.5 U/mL. Eggs were washed, then debris and unfertilized eggs or dead embryos were removed. The exposure was carried out using 6–well plates with 10 embryos in 3 mL of the control (E3 medium) or PS solution. In total, 10 embryos were exposed to and three replicates were run for each concentration. Prior to the study, the stock solution of the test compound was dissolved in E3 zebrafish embryo medium and then diluted to appropriate concentrations using this medium. The exposure solution was replaced every 24 h. Embryos were kept at 28 °C and in 14:10 light/dark conditions. The development of zebrafish embryos was monitored using a SteREO Discovery.V8 stereomicroscope (Zeiss, Oberkochen, Germany). The survival and hatching rates were examined at 24, 48, 72, and 96 hpf, while developmental abnormalities and heart rate (HR) values were examined at 48 and 72 hpf. Mortality was identified by missing heartbeat, coagulation of the embryos, failure to develop somites, and presence of a non–detached tail. In total, 10 embryos randomly selected from each concentration were used for morphological images. HR values of zebrafish embryos were counted by visual observation in 20–s intervals under the stereomicroscope.

### 2.4. hERG Channel Assay

The commissioned study was performed at the laboratory of SB Drug Discovery in Glasgow, United Kingdom. The inhibitory activity against hERG channels of PS at six concentrations (0.1, 0.4, 1.6, 6.25, 25, and 100 µg/mL) was assessed by automated patch clamp recordings using the SyncroPatch 384PE platform. The cell lines were generated by transfection of human embryonic kidney 293 cells with an expression vector encoding human hERG channels. A minimum five cells per concentrations were used. The concentrations were applied across the plate, with a single compound concentration per cell. The current was evoked using a peak pulse protocol from a holding potential of −90 mV to +25 mV over 5 s, followed by a 5 s repolarizing step at −50 mV, which was repeated every 15 s. The voltage protocol generation and data collection were performed with a software package consisting of PatchController384 V1.6.6 and Data Controller V1.8.0. Cisapride and H_2_O (vehicle) were added to the plate as positive and negative controls. The testing protocol consisted of three additions of extracellular physiological solution (4–7 min in total), then one concentration of the tested compound (2 min), followed by the reference channel blocker (1 min). Cells were removed if they exhibited identifiable solution exchange artifacts and an obvious loss in seal before the full block was applied. The % inhibition was calculated using the equation (1 − (I_conc_ − I_FB_)/(I_ref_ − I_FB_)), where I_conc_ was the current in the presence of the compound, I_FB_ was the current in the presence of the reference channel blocker, and I_ref_ was the current during the control period (in the absence of compound).

### 2.5. Cardiorespiratory Parameters up to 60 min after a Single Injection of Heparin and Protamine into Rats

**Figure 2:**
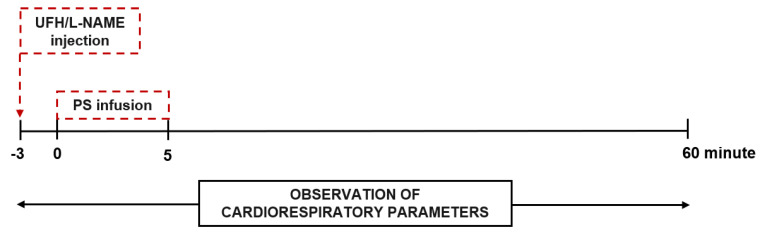
Schematic representation of cardiorespiratory parameters observation in rats treated with unfractionated heparin (UFH) and protamine sulfate (PS), and with N(ω)–nitro–l–arginine methyl ester (l–NAME) and PS.

Fifty–three male Wistar rats weighing 238.9 ± 26.8 g were randomly divided into 10 groups, anesthetized by intraperitoneal injection of pentobarbital (45 mg/kg), then placed in supine position on a heated operation table. UFH (150 and 1000 U/kg, 1 mL/kg) was administered into the right femoral vein alone, or a short–duration (5 min) intravenous infusion of PS (1.5 and 10 mg/kg, 1 mL/kg) was applied (2 mL/h) after UFH injection (Figure 2). l–NAME (25 mg/kg, 1 mL/kg) was administered into the right femoral vein alone, or a short–duration (5 min) intravenous infusion of PS (15 mg/kg, 1 mL/kg) was applied (2 mL/h) after l–NAME injection (Figure 2). The vehicle–treated (PBS, 1 mL/kg) animals served as the control group. HR, perfusion of paw vessels, blood oxygen saturation, respiratory rate, and peak exhaled CO_2_ values were measured using the PhysioSuite Physiological Monitoring Modular System (Kent Scientific Corporation, Torrington, CT, USA) for 60 min after drug administration. Mean blood pressure (MBP) was measured directly through a cannula filled with UFH solution (150 U/mL), placed in the left common carotid artery and connected to a pressure transducer (Plugsys, Transonics System Inc., Ithaca, NY, USA).

### 2.6. Histopathology of the Heart and Cardiac Troponin Measurement 35 Days after the Repeated (Once a Week) Injection of Heparin and Protamine into Mice

PS (1.5 mg/kg, 1 mL/kg) and UFH (150 U/kg, 1 mL/kg) alone or in combination were injected once weekly into the tail veins of 32 male BALB/c mice (26.0 ± 1.7 g) over five weeks. The vehicle–treated (PBS, 1 mL/kg) animals served as a control group. Blood samples were collected four times from each animal, who were anesthetized with a mixture of isoflurane and medical air (3% *v*/*v*) by puncture of the retro–orbital plexus on the day before drug administration. The blood samples were centrifuged at 8000 rpm at 22 °C for 5 min after 1 h of incubation at room temperature, then the serum samples were deep–frozen (−80 °C) until further determination of troponin concentration by immunoassay (R&D Systems, Minneapolis, MN, USA). No deaths, drug–related clinical signs of toxicity, effects on food consumption, or visual changes were reported over 35 days of observation. Blood samples were collected from the hearts under anesthesia (a mixture of isoflurane and medical air; 3% *v*/*v*) one week after administration of the last drug dose. Following the blood collection, the hearts were removed from mice and post–fixed in 4% formaldehyde. Then, they were cut on a Leica SM2000 R sliding microtome (Leica Biosystems, Nussloch, Germany) into 5 µm thick sections. Every tenth pair of sections was saved. The histological examination was conducted using hematoxylin and eosin staining under a Nikon Eclipse TE300 inverted microscope (Nikon Instruments Inc., Tokyo, Japan) equipped with an Olympus XC50 digital camera (Olympus Corporation, Tokyo, Japan) with the use of analySIS FIVE image analysis software for industrial microscopes.

### 2.7. Statistical Analysis

In the study, n refers to the number of animals in each experimental group. We chose the minimum necessary number of animals to detect the differences between each group based on our experience, as well as others’ experience using these procedures. The data are shown as the median with the range or mean with the SEM. All the datasets were tested for normality using the Shapiro–Wilk test. Kruskal–Wallis ANOVA with Dunn’s post–hoc test was used to analyze data with non–Gaussian distributions. The survival data were evaluated using the log–rank test. The results were analyzed and graphically presented using GraphPad Prism 8 (GraphPad Software, La Jolla, CA, USA). Here, *p* values < 0.05 were considered significant.

## 3. Results

### 3.1. The Effects of Protamine on Zebrafish Embryo Development and Cardiac Function during Chronic and 48-h Exposure

**Figure 3:**
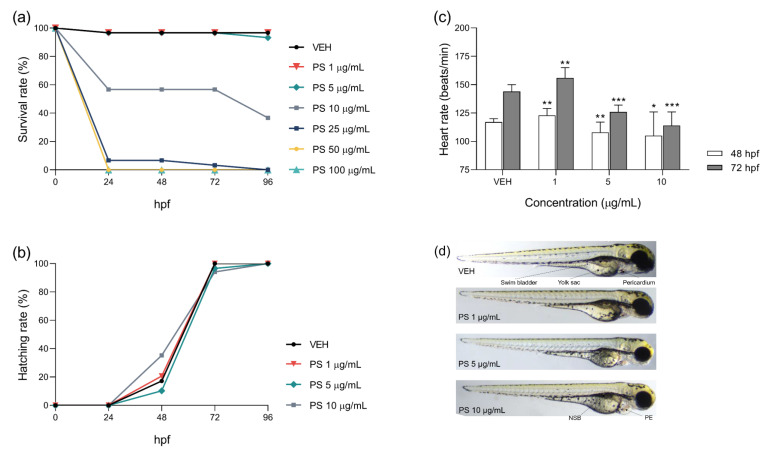
Survival (**a**) and hatching (**b**) rates for zebrafish embryos exposed to protamine sulfate (PS) concentrations or vehicle (VEH) from the 2nd to 96th hour post–fertilization (hpf). Heart rate (**c**) values of zebrafish embryos and larvae at 48 and 72 hpf. Morphology (**d**) of zebrafish larvae at 72 hpf. Abbreviations: NSB, non–inflated swim bladder; PE, pericardial edema. Data are shown as medians with ranges. Note: * *p* < 0.05; ** *p* < 0.01, *** *p* < 0.001 vs. VEH within the group, Kruskal–Wallis ANOVA with Dunn’s post–hoc test, *n* = 17–29 for each concentration.

Survival and hatching rates were examined at 24, 48, and 72 hpf during chronic exposure to PS. The range of PS concentrations used from 1 to 100 µg/mL reflects the levels achieved during neutralization of UFH and when PS is overdosed [21,22]. Significant and concentration–dependent lethality was observed before 24 hpf. PS added at concentrations of 1 and 5 µg/mL did not cause significant embryo death throughout the assay. Survival was lower at 10 and 25 µg/mL of PS when compared to vehicle (*p* < 0.001), while PS concentrations of 50 and 100 µg/mL caused immediate death of 100% of the embryos (*p* < 0.001) (Figure 3a). Almost all of the embryos in the tested groups hatched at 72 hpf (Figure 3b). PS concentrations of 25, 50, and 100 µg/mL were not included in Figure 3b due to 100% mortality. PS at a concentration of 10 µg/mL induced obvious cardiac defects such as pericardial edema and non–inflated swim bladder of zebrafish larvae at 72 hpf (Figure 3d). We did not investigate the HR values of embryos incubated with PS at concentrations of 25, 50, or 100 µg/mL because the lethality was too high. As shown in Figure 3c, exposure to PS at a concentration of 1 µg/mL significantly affected the cardiac function of zebrafish embryos and larvae by increasing HR compared to the control group at 48 and 72 hpf. However, the addition of PS at higher concentrations significantly decreased HR in a concentration–dependent manner.

**Figure 4:**
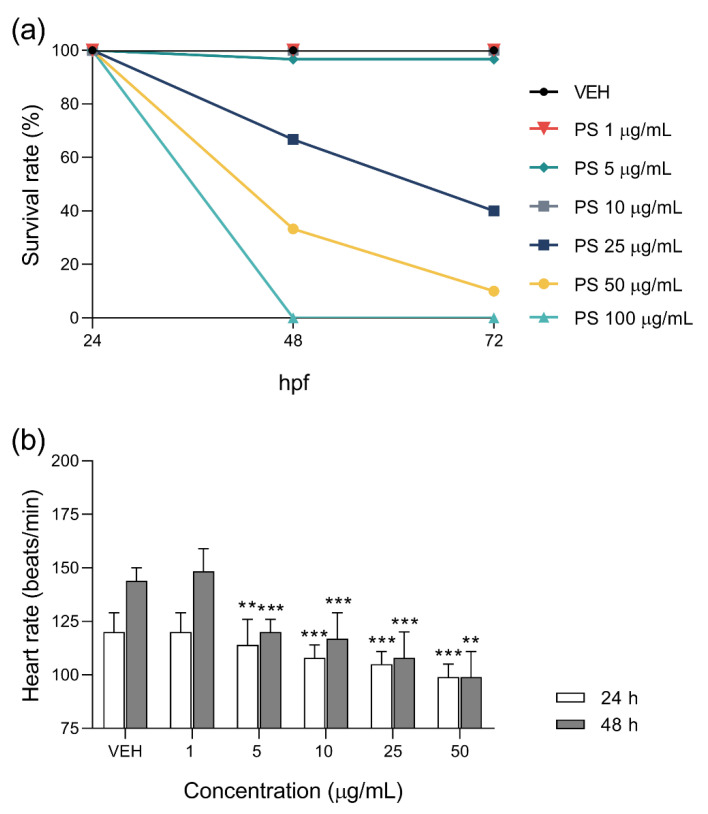
Survival (**a**) and heart (**b**) rates of zebrafish embryos at 24 h post–fertilization (hpf) exposed for 24 and 48 h to vehicle (VEH) and protamine sulfate (PS) concentrations. Data are shown as medians with ranges. Note: ** *p* < 0.01, *** *p* < 0.001 vs. VEH within the group, Kruskal–Wallis ANOVA with Dunn’s post–hoc test, *n* = 29–30 for VEH group and for PS at concentrations of 1, 5, and 10 µg/mL; *n* = 3–20 in PS at concentrations for 25 and 50 µg/mL.

The mortality of 24 hpf zebrafish embryos exposed to PS for 24 and 48 h increased in a concentration–dependent manner and was found to be lower than during chronic exposure. PS at concentrations of 1 to 10 µg/mL did not significantly reduce embryo survival. The survival rates of embryos incubated with PS at concentrations of 25, 50, and 100 µg/mL at 72 hpf were 40%, 10%, and 0%, respectively (*p* < 0.001) (Figure 4a). PS at a concentration of 1 µg/mL did not affect HR, but at a concentration of 5 µg/mL caused a significant decrease in HR, which further decreased at higher concentrations (Figure 4b).

### 3.2. The Effects of Protamine, l–NAME, and Heparin on Cardiac Function of Zebrafish Embryos during 48–Hours Exposure

**Figure 5:**
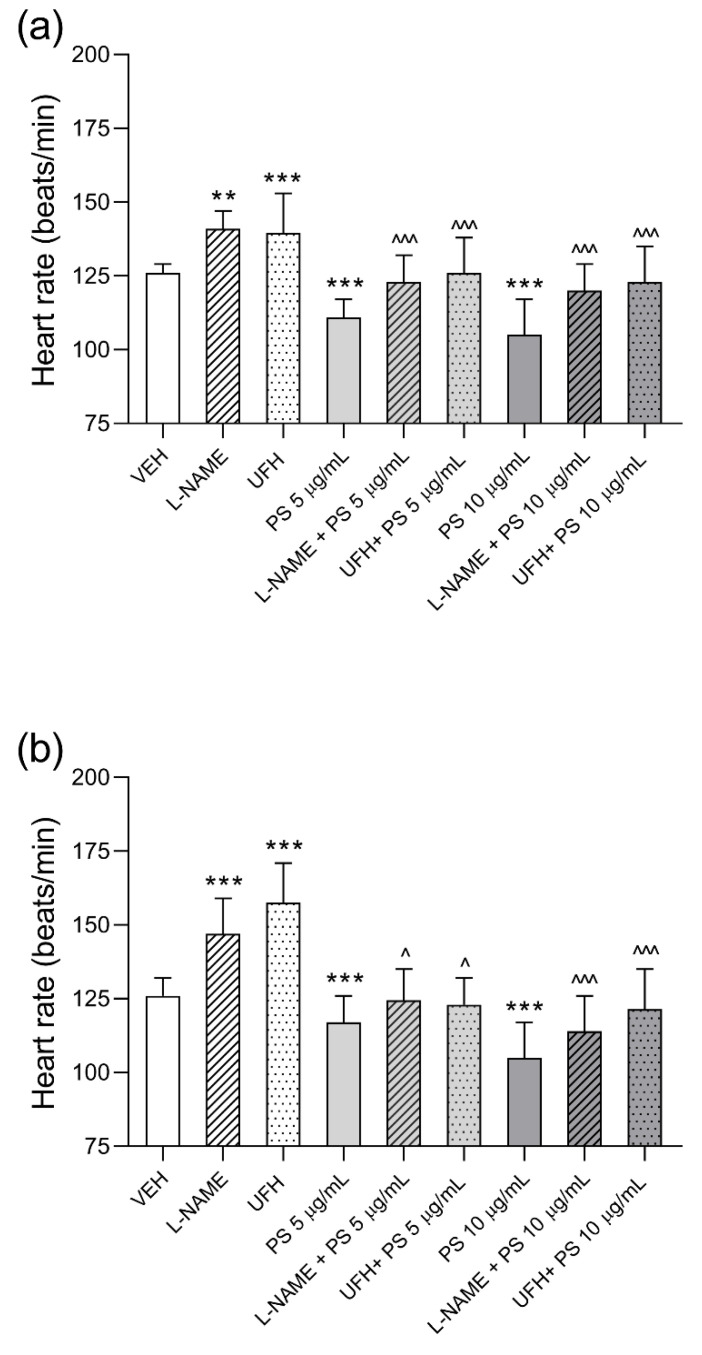
Heart rate values of zebrafish embryos at 24 h post–fertilization exposed for 24 (**a**) and 48 h (**b**) to vehicle (VEH), l–NAME (0.5 mM), unfractionated heparin (UFH, 0.5 U/mL), protamine sulfate (PS), PS together with l–NAME (0.5 mM), or PS together with UFH (0.5 U/mL). Data are shown as medians with ranges. Note: ** *p* < 0.01, *** *p* < 0.001 vs. VEH, ^ *p* < 0.05 ^^^ *p* < 0.001 vs. PS at the appropriate concentration, Kruskal–Wallis ANOVA with Dunn’s post–hoc test, *n* = 30 for each concentration.

HR was significantly higher in zebrafish embryos exposed to 24 and 48 h of N(ω)–nitro–l–arginine methyl ester (l–NAME) and UFH compared to the control group. PS, similarly to previous experiments, significantly decreased HR at concentrations of 5 and 10 µg/mL (Figure 4b). l–NAME incubated together with PS for 24 and 48 h with zebrafish embryos completely abolished PS effects on HR (Figure 5a,b). The application of PS and UFH in mass ratios of 1:1 and 2:1 returned the values to control levels (Figure 5b).

### 3.3. The Acute Effects of Protamine and Its Complexes with Heparin on Blood Pressure, Cardiac Function, and Respiratory Function in Rats

**Figure 6:**
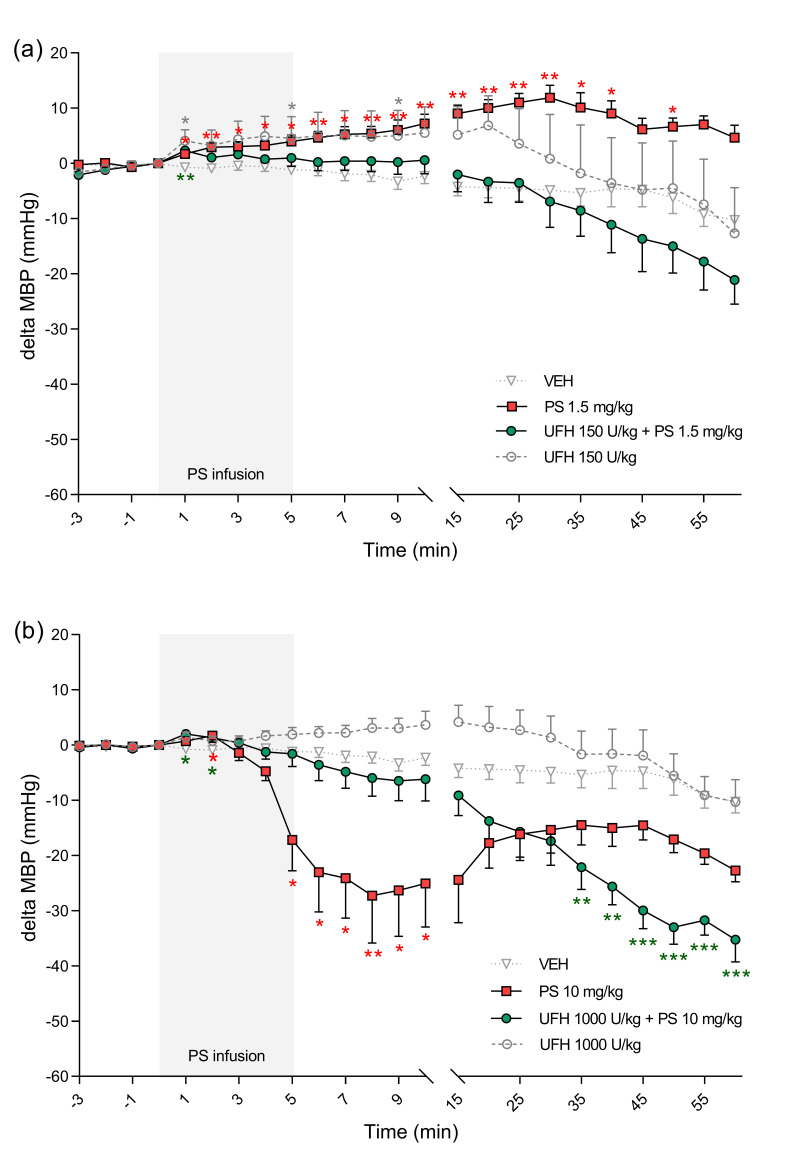
The effects of protamine sulfate (PS) and its complexes with unfractionated heparin (UFH) on mean blood pressure (MBP) values. The course of MBP was tracked for 60 min after short–duration intravenous infusion (5 min; 2 mL/h) of PS at a dose of 1.5 (**a**) or 10 mg/kg (**b**) following intravenous administration of vehicle (VEH, 1 mL/kg) or UFH (150 or 1000 U/kg, 1 mL/kg). Results are shown as means (lines) with SEMs. The MBP values at 0 min of the experiment were 121.4 (86.6–138.0) for VEH, 110.7 (91.1–129.4) for PS 1.5 mg/kg, 134.6 (122.8–152.7) for UFH 150 U/kg + PS 1.5 mg, 109.4 (93.1–127.4) for UFH 150 U/kg, 116.9 (108.2–135.6) for PS 10 mg/kg, 120.3 (93.5–130.8) for UFH 1000 U/kg + PS 10 mg/kg, 107.7 (95.2–126.6) for UFH 1000 U/kg. Note: * *p* < 0.05, ** *p* < 0.01, *** *p* < 0.001 vs. VEH, Kruskal–Wallis ANOVA with Dunn’s post–hoc test, *n* = 5–8.

**Figure 7:**
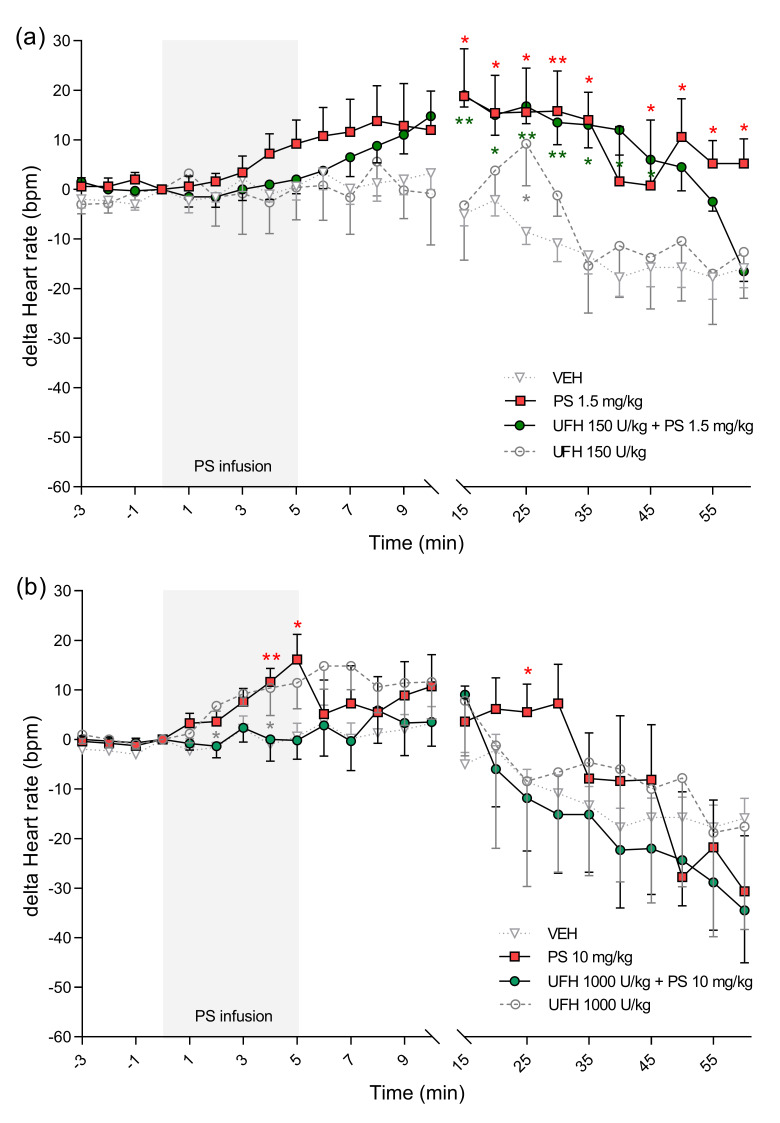
The effects of protamine sulfate (PS) and its complexes with unfractionated heparin (UFH) on heart rate. The course of heart rate was tracked for 60 min after short–duration intravenous infusion (5 min; 2 mL/h) of PS at a dose of 1.5 (**a**) or 10 mg/kg (**b**) following intravenous administration of vehicle (VEH, 1 mL/kg) or UFH (150 or 1000 U/kg, 1 mL/kg). Results are shown as means (lines) with SEMs. The heart rate values at 0 min of the experiment were 438 (365–488) for VEH, 407 (364–438) for PS 1.5 mg/kg, 437 (388–458) for UFH 150 U/kg + PS 1.5 mg, 416 (345–476) for UFH 150 U/kg, 424 (378–517) for PS 10 mg/kg, 410 (385–432) for UFH 1000 U/kg + PS 10 mg/kg, 390 (308–442) for UFH 1000 U/kg. Note: * *p* < 0.05, ** *p* < 0.01 vs. VEH, Kruskal–Wallis ANOVA with Dunn’s post–hoc test, *n* = 5–8.

PS alone and in the complexes with UFH significantly changed cardiorespiratory parameters in rats. PS administered at a dose of 1.5 mg/kg increased MBP and HR (Figure 6a and Figure 7a). PS administered at a dose of 10 mg/kg led to significant decreases of MBP between the 5th and 10th minute maximally by 23% 8 min after starting of infusion. The same PS dose administered with UFH caused an even more profound drop of MBP, starting at the 35th minute and reaching a maximum 29% decrease at the end of the experiment (Figure 6b).

**Figure 8:**
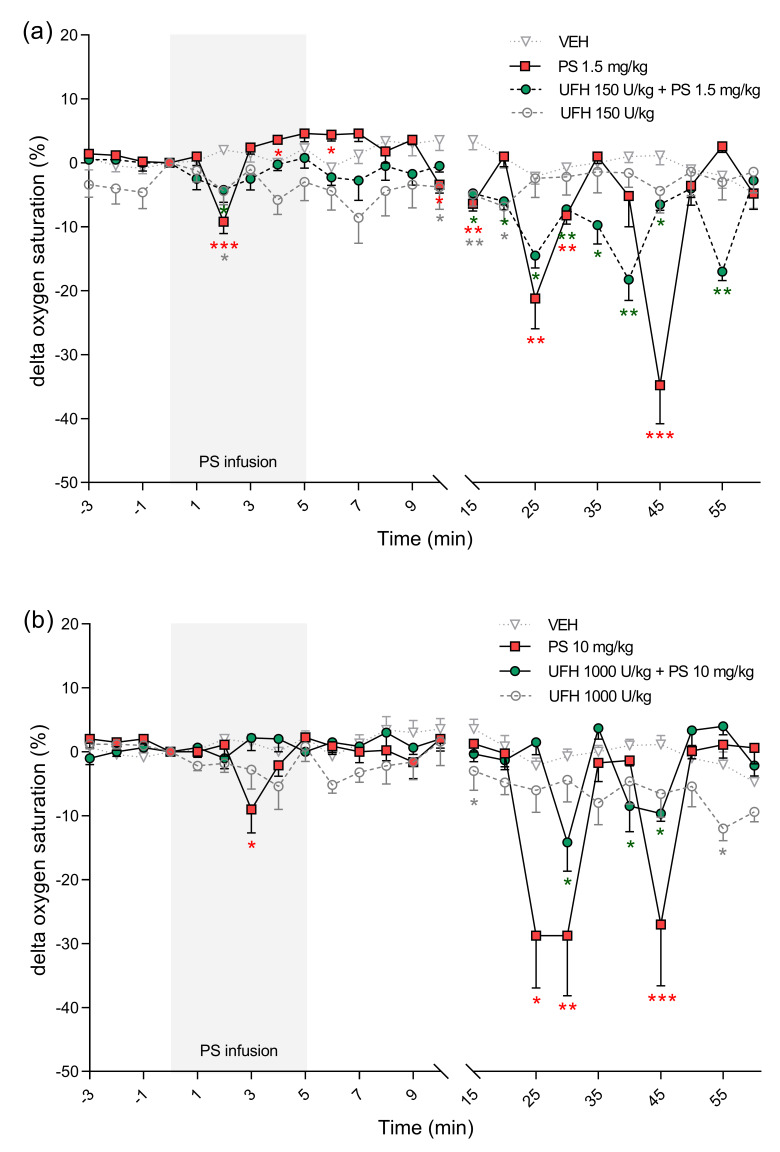
The effects of protamine sulfate (PS) and its complexes with unfractionated heparin (UFH) on blood oxygen saturation. The course of blood oxygen saturation was tracked for 60 min after short–duration intravenous infusion (5 min; 2 mL/h) of PS at a dose of 1.5 (**a**) or 10 mg/kg (**b**) following intravenous administration of vehicle (VEH, 1 mL/kg) or UFH (150 or 1000 U/kg, 1 mL/kg). Results are shown as means (lines) with SEMs. The blood oxygen saturation values at 0 min of the experiment were 82 (72–89) for VEH, 82 (73–86) for PS 1.5 mg/kg, 85 (81–87) for UFH 150 U/kg + PS 1.5 mg, 85 (84–94) for UFH 150 U/kg, 79 (73–86) for PS 10 mg/kg, 79 (72–89) for UFH 1000 U/kg + PS 10 mg/kg, 87 (80–99) for UFH 1000 U/kg. Note: * *p* < 0.05, ** *p* < 0.01; *** *p* < 0.001 vs. VEH, Kruskal–Wallis ANOVA with Dunn’s post–hoc test, *n* = 5–8.

**Figure 9:**
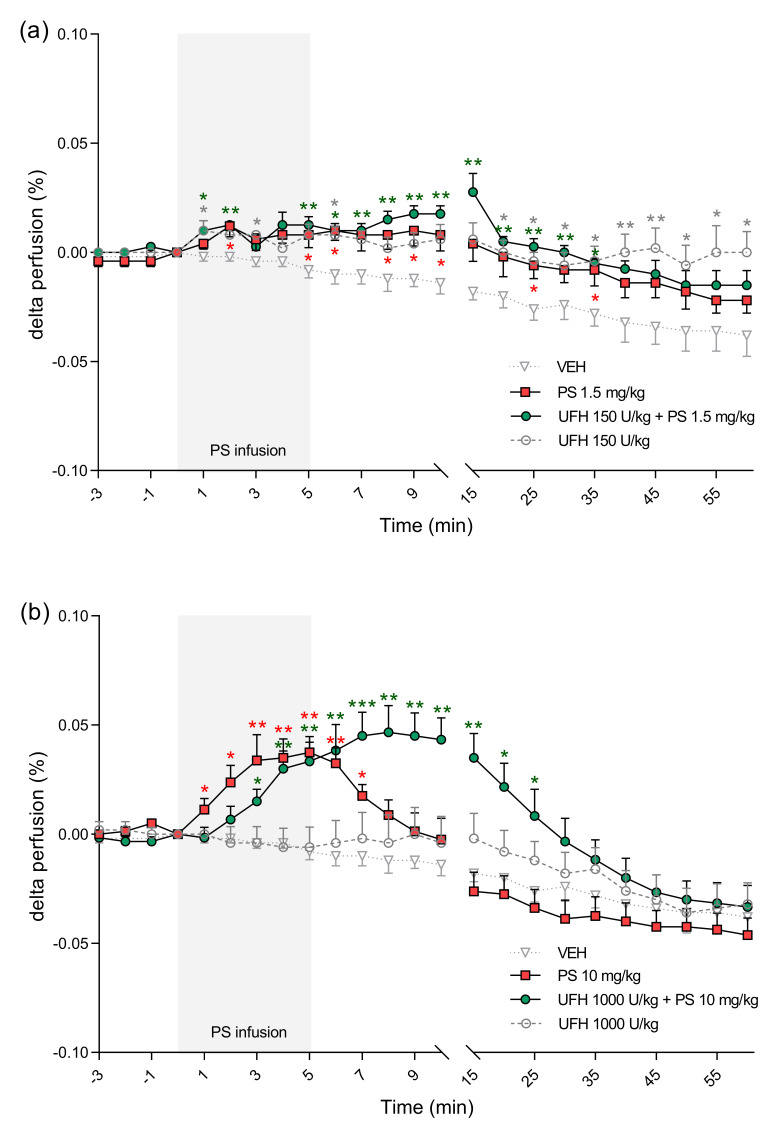
The effects of protamine sulfate (PS) and its complexes with unfractionated heparin (UFH) on perfusion of paw vessels. The course of perfusion of paw vessels was tracked for 60 min after short–duration intravenous infusion (5 min; 2 mL/h) of PS at dose of 1.5 (**a**) or 10 mg/kg (**b**) following intravenous administration of vehicle (VEH, 1 mL/kg) or UFH (150 or 1000 U/kg, 1 mL/kg). Results are shown as means (lines) with SEMs. The perfusion of paw vessels at 0 min of the experiment were 0.08 (0.04–0.14) for VEH, 0.07 (0.05–0.11) for PS 1.5 mg/kg, 0.07 (0.02–0.13) for UFH 150 U/kg + PS 1.5 mg, 0.06 (0.03–0.1) for UFH 150 U/kg, 0.09 (0.05–0.13) for PS 10 mg/kg, 0.1 (0.06–0.14) for UFH 1000 U/kg + PS 10 mg/kg, 0.08 (0.05–0.13) for UFH 1000 U/kg. * *p* < 0.05, ** *p* < 0.01, *** *p* < 0.001 vs. VEH, Kruskal–Wallis ANOVA with Dunn’s post–hoc test, *n* = 5–8.

**Figure 10:**
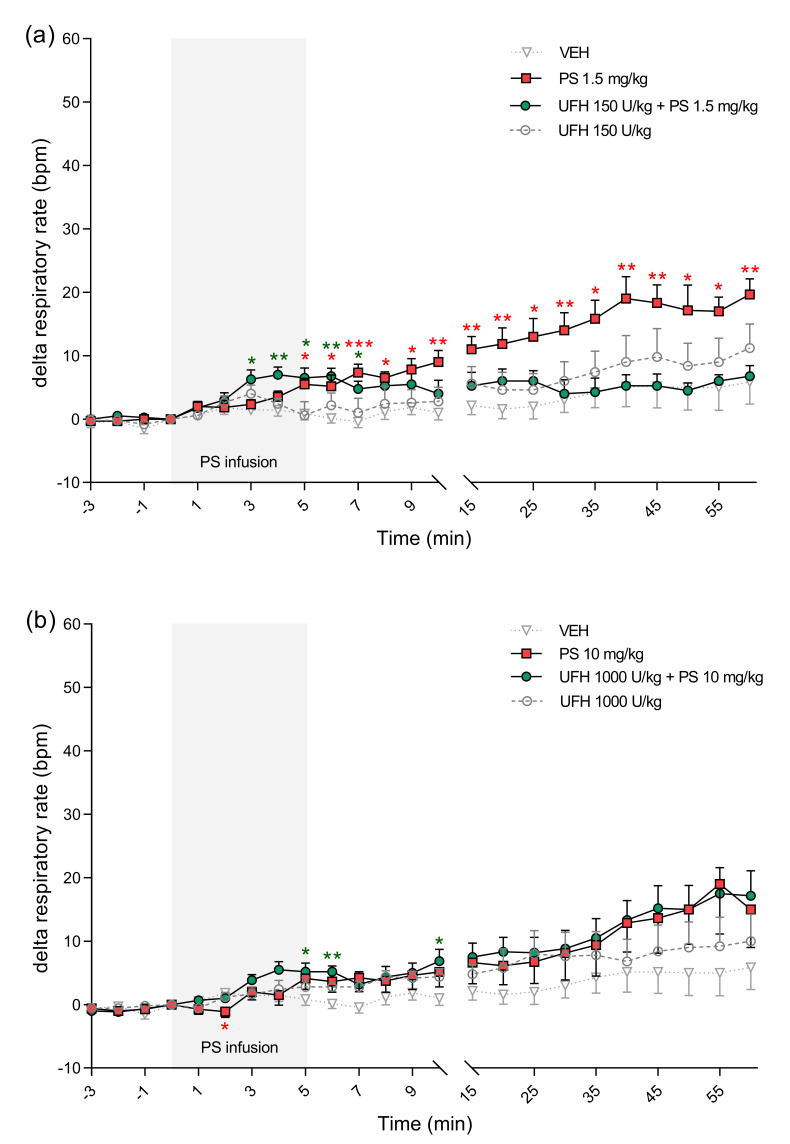
The effects of protamine sulfate (PS) and its complexes with unfractionated heparin (UFH) on respiratory rate. The course of respiratory rate was tracked for 60 min after short–duration intravenous infusion (5 min; 2 mL/h) of PS at dose of 1.5 (**a**) or 10 mg/kg (**b**) following intravenous administration of vehicle (VEH, 1 mL/kg) or UFH (150 or 1000 U/kg, 1 mL/kg). Results are shown as means (lines) with SEMs. The respiratory rate at 0 min of the experiment were 68 (43–88) for VEH, 68 (63–70) for PS 1.5 mg/kg, 76 (65–83) for UFH 150 U/kg + PS 1.5 mg, 64 (50–75) for UFH 150 U/kg, 67 (48–90) for PS 10 mg/kg, 67 (40–95) for UFH 1000 U/kg + PS 10 mg/kg, 52 (41–59) for UFH 1000 U/kg. Note: * *p* < 0.05, ** *p* < 0.01, *** *p* < 0.001 vs. VEH, Kruskal–Wallis ANOVA with Dunn’s post–hoc test, *n* = 5–8.

**Figure 11:**
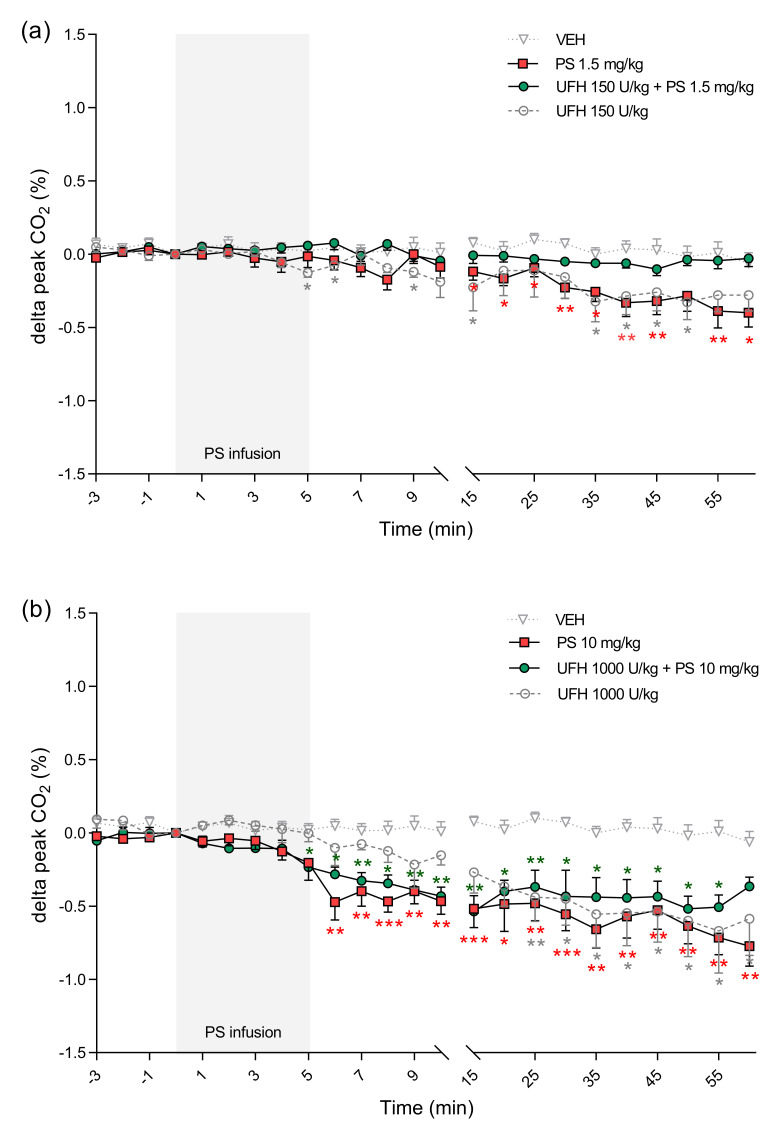
The maximum effects of protamine sulfate (PS) and its complexes with unfractionated heparin (UFH) on peak exhaled CO_2_. The course of peak exhaled CO_2_ was tracked for 60 min after short–duration intravenous infusion (5 min; 2 mL/h) of PS at dose of 1.5 (**a**) or 10 mg/kg (**b**) following intravenous administration of vehicle (VEH, 1 mL/kg) or UFH (150 or 1000 U/kg, 1 mL/kg). Results are shown as means (lines) with SEMs. The peak exhaled CO_2_ values at 0 min of the experiment were 4.2 (4.0–4.7) for VEH, 4.1 (4.0–4.2) for PS 1.5 mg/kg, 4.0 (3.7–4.4) for UFH 150 U/kg + PS 1.5 mg, 4.0 (3.5–4.8) for UFH 150 U/kg, 4.2 (3.6–5.0) for PS 10 mg/kg, 4.5 (4.2–5.0) for UFH 1000 U/kg + PS 10 mg/kg, 4.6 (3.9–5.1) for UFH 1000 U/kg. Note: * *p* < 0.05, ** *p* < 0.01, *** *p* < 0.001 vs. VEH, Kruskal–Wallis ANOVA with Dunn’s post–hoc test, *n* = 5–8.

PS at lower doses alone or in combination with UFH increased HR (Figure 7a). However, it decreased HR by 7% when administered at higher doses (Figure 7b). We found decreases in blood oxygen saturation in all groups, however in rats treated with PS at higher dose alone the changes were the most significant with peak drops at the 30th minute by 37% and at the 45th minute by 34% (Figure 8b). PS administered at a dose of 10 mg/kg induced an immediate significant increase in perfusion of paw vessels maximally by 33%, lasting 6 min (Figure 9b). The effects recorded in rats treated with UFH and PS were delayed and lasted longer from the 3rd to 25th minute, with a maximum increase of 50% (Figure 9b). Higher dose of PS tended to increase the respiratory rate by 29% (Figure 10b) and decreased peak CO_2_ by 19% (Figure 11b) at the end of experiment. We observed similar effects in groups treated with higher doses of PS and UFH, although changes in respiratory parameters were less severe (Figure 10b and Figure 11b). A progressive increase in respiration rate coincided with a decrease in peak CO_2_ in the group treated with a low dose of PS (Figure 10a and Figure 11a).

UFH administered at doses of 150 U/kg and 1000 U/kg did not provoke significant changes in any cardiorespiratory parameters. It had no effect on MBP, HR, or respiratory rate (Figure 6a,b, Figure 7a,b and Figure 10a,b). UFH at both doses decreased blood oxygen saturation and peak CO_2_, but not as severely as PS (Figure 8a,b and Figure 11a,b). UFH at a lower dose slightly increased perfusion of paw vessels at the end of the experiment (Figure 9a).

### 3.4. The Involvement of NO in Protamine–Induced Hypotension in Rats

**Figure 12:**
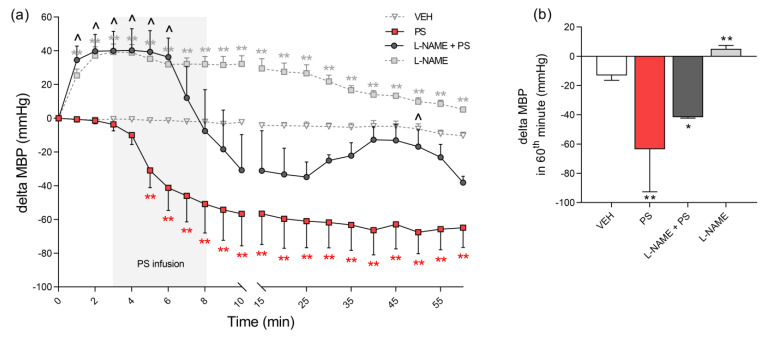
The effects of protamine sulfate (PS) alone and together with N(ω)–nitro–l–arginine methyl ester (l–NAME) on mean blood pressure (MBP). The course of MBP was tracked for 60 min after short–duration intravenous infusion (5 min; 2 mL/h) of PS (15 mg/kg) following intravenous administration of vehicle (VEH; 1 mL/kg) or l–NAME (25 mg/kg; 1 mL/kg). Results are shown as means (line) with SEMs (**a**) and as medians with ranges (**b**). The MBP values at 0 min of the experiment were 121.4 (86.6–138.0) for VEH, 139.3 (130.4–153.3) for PS 15 mg/kg, 124.9 (107.1–119.8) for PS 10 mg/kg + l–NAME, and 132.5 (118.5–138.9) for l–NAME alone. Note: * *p* < 0.05, ** *p* < 0.01 vs. VEH, ^ *p* < 0.05 vs. PS 15 mg/kg, Kruskal–Wallis ANOVA with Dunn’s post–hoc test, *n* = 3–5.

PS induced immediate significant hypotension compared to the control group, lasting to the end of the experiment. MBP dropped maximally by 49% at the 50th minute (Figure 12a). l–NAME administrated 3 min before PS infusion reduced the hypotensive effect by 42% (Figure 12b).

### 3.5. The Chronic Effects of Protamine and Its Complexes with Heparin on Cardiac Biomarkers in Mice

**Figure 13:**
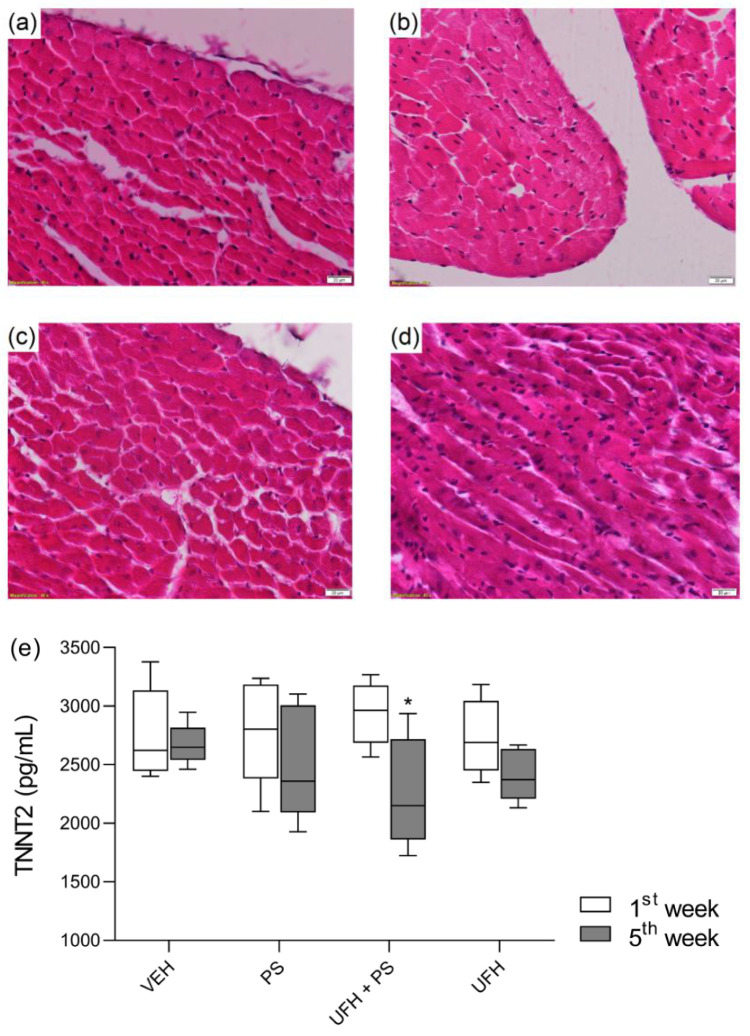
Histopathology of the heart tissue samples from mice treated five times with vehicle (VEH, 1 mL/kg) (**a**), protamine sulfate alone (PS, 1.5 mg/kg, 1 mL/kg) (**b**), or with unfractionated heparin (UFH, 1.5 U/kg, 1 mL/kg) (**c**) and only UFH (1.5 U/kg, 1 mL/kg) (**d**). The concentrations of cardiac troponin T type 2 (TNNT2) in mice serum in the first week after a single administration and in the 5th week after four administrations of drugs (**e**). The results are shown as the medians (line) with the interquartile ranges (box), along with maximum and minimum values (whiskers). Note: * *p* < 0.05 vs. VEH within the group, Kruskal–Wallis ANOVA with Dunn’s post–hoc test, *n* = 6–8.

Histological analysis of cardiac muscle revealed that all sections of tested hearts had a normal morphological structure in every group. We observed samples of the visceral pericardium covered with the mesothelium, the cells of which had the correct appearance. Myocytes were of cylindrical shape and were connected to each other by cellular bridges. Muscle fibers showed a striated structure. Between them, we were able to see blood vessels, sometimes branching into smaller ones. Myocardial cells usually had one nucleus and sometimes two nuclei, which were not enlarged. Neither necrotic tissue nor pathological lesions were observed. Annuli around all valves were formed by a regular fibrous skeleton of the heart. The connective tissue stretched from the skeleton into the valves and further into chordae tendineae and papillary muscle. Valves showed an unchanged structure with three distinguishable layers covered with endothelia. Valves were of a bluish color specific for hematoxylin and eosin staining. No calcification was observed. The endocardium did not show abnormal features (Figure 13a–d).

The concentrations of serum cardiac troponin T type 2 (TNNT2), a biomarker of cardiac injury, did not rise significantly in the first week after a single administration or in the 5th week after four administrations of PS alone or PS with UFH. We observed only a slight decrease in TNNT2 levels after repeated administration of UFH and PS together (Figure 13e).

### 3.6. The Effects of Protamine on hERG Channel Activity

**Figure 14:**
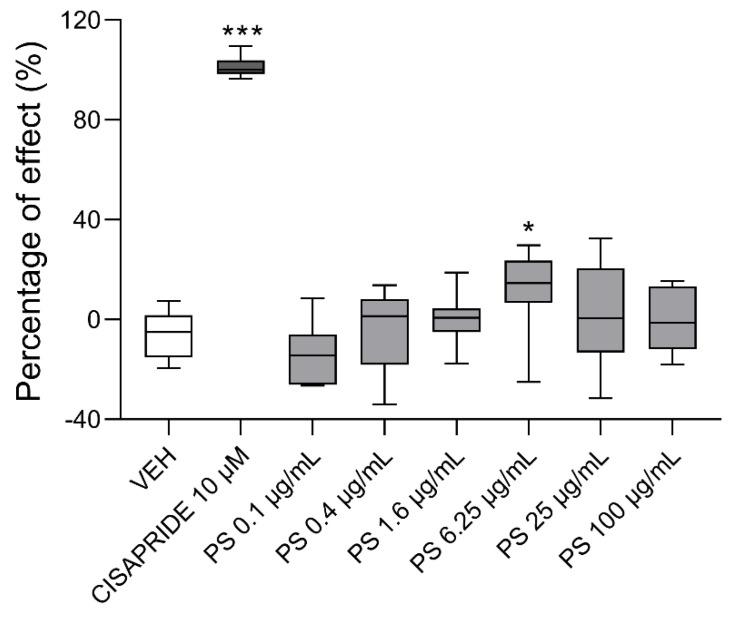
The effects of protamine sulfate (PS), vehicle (VEH), and cisapride on hERG K^+^ currents. Data are shown as medians (line) with interquartile ranges (box) and maximum and minimum values (whiskers). Note: * *p* < 0.05, *** *p* < 0.001 vs. VEH, Kruskal–Wallis ANOVA with Dunn’s post–hoc, *n* = 6–15 in each concentration.

The electrophysiological profiling of PS was performed at six concentrations (0.1, 0.4, 1.6, 6.25, 25, and 100 µg/mL) on the hERG channels. Figure 14 displays the percentages of PS effects at the tested concentration and for positive (cisapride) and negative (H_2_O) controls. Cisapride at a concentration of 10 µM completely blocked the hERG channels. Only PS at 6.25 µg/mL showed a slight inhibitory effect on the hERG currents. Since PS did not induce concentration–dependent inhibition of the channels, we were unable to calculate the half maximal inhibitory concentration values.

## 4. Discussion

We have shown that the circulatory and respiratory systems are among the targets of PS toxicity. PS affected the heart function of embryos and larvae of zebrafish, increasing their mortality rates at higher concentrations and with longer durations of incubation. Adverse cardiovascular effects of PS observed in rats were also more pronounced at higher dose. Changes in MBP, HR, and vascular perfusion were accompanied by the disturbances in respiratory system, manifested by increases in respiratory rate and decreases in exhaled CO_2_ and blood oxygen saturation. The bradycardia in zebrafish embryos and larvae and the hypotension in rats were partially attenuated by l–NAME, indicating the involvement of NO. In general, polycationic PS affected the tested parameters more significantly than its neutral complexes with UFH in rats and zebrafish embryos and larvae. Thus, we may conclude that PS induces cardiorespiratory toxicity, which is charge–dependent and involves enhanced release of NO. We provide here clear evidence of the acute cardiorespiratory toxicity of PS in zebrafish and rats, although we found no direct arrhythmogenic activity in human cell culture or signs of permanent long–term damage of heart tissue caused by PS in mice.

Because of differences in the physiological responses to UFH and PS between humans and animals, we chose the doses based on the comparable anticoagulant effects of UFH in humans and in rats. Since UFH at a dose of 150 U/kg extended activated partial thromboplastin time by almost 3 times in rats [23], based on clinical guidelines where 1 mg of protamine neutralizes 100 U of UFH [24], we chose 1.5 mg/kg as a starting dose. We further increased the dose of PS to 10 mg/kg, which is still a well–tolerated intravenous dose in rats according to the literature [25], so as to observe the escalation of potential side effects. The 5–50 µg/mL range of PS concentrations in zebrafish and human hERG channels reflects its concentrations achieved in patients after injection of 0.5 mg/kg–250 mg, whereas a concentration of 100 µg/mL reflects overdosing of PS [21,22].

We used zebrafish, which are prominent model vertebrate organisms, for developmental and toxicological studies of new drug candidates. Zebrafish are especially useful for studying cardiotoxicity because cardiac abnormalities do not cause immediate death of zebrafish embryos [26]. Therefore, we were able to use higher PS concentrations, which would be lethal in rodents and cause significant suffering. As far as we know, the effects of PS and UFH on zebrafish observed herein are the very first to be published. Our main challenge was the route of PS administration, which being a charged polypeptide, had to penetrate the interiors of fish embryos and larvae. PS can pass across the cellular membrane and can promote penetration of associated compounds through biological barriers. These properties have been widely investigated for different drug delivery applications [27]. The chorion is the first barrier that PS had to overcome during the incubation with zebrafish before hatching (48 hpf). This is a cellular barrier in which the transport of molecules can only occur through diffusion. The studies showed that nanocapsules with double shells of hyaluronic acid and PS crossed the chorion and could be transported through the epidermis in hatched embryos. It is possible that PS, as a membrane–translocating peptide, could facilitate the transport through protein and epithelial barriers of zebrafish [27]. Although we found increased mortality after the incubation of zebrafish embryos with PS at concentrations above 10 µg/mL, harmful effects in survivors were moderate. The others reported that cationic polymers with similar molecular weights to PS induced 50% mortality at concentrations below 1 µg/mL [28]. Poly(ethylene imine)–based cationic polymers caused approximately 50% mortality of zebrafish embryos at a concentration of 5 µg/mL [29]. Nevertheless, these compounds reduced HR in a concentration–dependent manner and induced pericardial edema similarly to PS [29]. Additionally, we showed that anionic UFH at an appropriate weight ratio to cationic PS was able to attenuate the decrease in HR caused by PS in zebrafish embryos and larvae. The electrostatic charge of the drugs tested may have profound effects on zebrafish heart function. Our results and literature data suggest that the positive charge is at least partially responsible for the toxicity of PS, leading to death of immature living organisms. Our results also indicate that compounds with similar chemical structures to UFH may increase HR, and testing their toxicity in a zebrafish model may cause interference from the cardiovascular system.

Cardiac arrest has recently been reported for the first time after the slow infusion of PS in three clinical cases [16]. The patients, who had never before had episodes of hypersensitivity, respiratory disfunction, or insulin–dependent diabetes, experienced profound hypotension followed by ventricular fibrillation within minutes of the administration of PS. One of them had symptoms of pulmonary vasoconstriction and hypertension with acute right heart dilatation. This could be explained by prolonged cardiac arrest, however PS was also suggested as a possible cause. Animal studies have shown that PS may directly suppress myocardial contractility and may influence the vascular system [30]. In our study, PS increased HR in both zebrafish embryos and larvae at low concentrations, although the effects were significant only after chronic exposure. The incubation with PS at increasing concentrations resulted in progressive decrease of HR accompanied by pericardial edema. This suggests that these changes might also occur in mammals. However, we did not find an increase of troponin level and any histopathological signs of heart tissue permanent damage after single or repeated administration of PS alone or with UFH in mice. In contrast, we observed a slight decrease in troponin level in mice treated with UFH and PS, which could be explained by electrostatic binding of cardiac protein by UFH [31]. Simultaneous initial increase of HR observed in rats could also be a part of the natural physiological response of the circulatory system to decrease of MBP. Undoubtedly, the heart is a target of PS acute toxicity, however the arrhythmogenic effect of PS is poorly supported by the literature. The evaluation of the ability to block hERG channel currents is the method recommended to assess cardiac safety in drug development [32]. These channels are responsible for the critical current in the repolarization of cardiac action potential, the rapid delayed rectifier K^+^ current, and their alterations have been related to arrhythmia [33,34]. Except for the very low, negligible block of hERG channels at the concentration of 6.25 µg/mL, PS did not change the amplitude of rapid delayed rectifier K^+^ current. Other studies have shown that isolated rat cardiac muscle under the influence of PS behaved as it did in the presence of calcium channel blockers, reducing contractility in a dose–dependent manner [35]. Perhaps PS can modify only cellular calcium shifts via sarcolemmal ion channels, which could promote ventricular arrhythmia. The ventricular fibrillation could also be a consequence of myocardial hypoperfusion arising from systemic arterial hypotension [16]. In our study, PS affected HR in zebrafish and rats more strongly than its complexes with UFH, pointing to PS as a key factor.

Systemic hypotension may result from the actions of vasoconstricting and vasorelaxing mediators released by PS [2,5]. Wakefiled et al. suggested that PS can exert two independent hypotensive effects in heparinized dogs. The first is most likely due to direct vasodilatory activity, in addition manifested by a decline in systemic oxygen consumption [19]. The second hypotensive effect is probably related to the accumulation and aggregation of platelets in the lungs with the release of vasoconstrictive mediators there [19]. l–Arginine, the main component of PS, is the physiological precursor of NO, an endogenous vasodilator formed during the oxidative deamination by NO synthase (NOS) [5]. The pretreatment of isolated human and rabbit arteries with a competitive inhibitor of endothelial NOS almost completely blocked PS–induced vasorelaxation [36,37]. Therefore, we decided to verify whether NO is involved in PS cardiovascular effects. PS induced an immediate hypotension in rats and l–NAME partially blocked this effect. Simultaneously, we observed the increase in perfusion of paw vessels, which also points to vasodilatation. NO plays an important function in the cardiovascular system, not only in mammals but also in fish [38,39]. NOS was detected in endothelial cells of dorsal veins and in the hearts of zebrafish larvae. At these stages of development, the heart has only primitive atrioventricular cushions without functional autonomic innervation. The application of NO donors such as sodium nitroprusside and isosorbide dinitrate to zebrafish larvae and rainbow trout alevins resulted in significant vasodilatation and a decrease of HR. l–NAME exerted opposite effects [38,40]. Similarly, we found that l–NAME increased HR, but also completely abolished the effects of PS after 24 and 48 h of exposure in embryonic and larval zebrafish, possibly by competing with l–arginine released from PS. We found that MBP decreased during 5 min infusion of PS alone into rats, and when PS was complexed with UFH the hypotensive effect was delayed. It is possible that UFH, through its anionic groups, inhibits the interactions of cationic groups of PS with endothelial or blood cells. Otherwise, the cationic nature of PS could stimulate the release of different mediators, such as NO, bradykinin, histamine, or cyclooxygenase metabolites of arachidonic acid, and thus activate membrane receptors and ion channels [2,5]. Wakefield et al. showed that a decrease of MBP after PS was accompanied by thrombocytopenia [20]. UFH and PS complexes interacting with platelets and leukocytes may release histamine [10,11] or thromboxane [12,13,41], leading to pulmonary vasoconstriction, then to pulmonary hypertension with subsequent edema, finally causing systemic hypotension. This part of the event sequence was observed in animals [3,20] and occasionally in patients [15], but the exact mechanism was never confirmed [2,10,15]. We have recently showed that both PS alone and together with UFH did not induce thrombocytopenia, platelet activation, or aggregation in rats and mice. On the contrary, PS inhibited platelet aggregation [42]. The others showed that blockers of vasoconstrictive mediators released from platelets only partially restored normal hemodynamic and respiratory parameters in animal models and patients [13,41,43,44]. The partial inhibition of PS–induced hypotension by l–NAME together with the PS antiplatelet effects previously demonstrated by us indicate the involvement of short–acting mediators such as NO in the mechanism of action [42]. The delay of hypotensive effect of PS by the neutralization with UFH may also be a result of differences in the metabolism of PS or the short action of NO. Akata et al. observed that UFH prevented vasodilation of isolated human small arteries induced by PS [45], while in another study it did not [36]. A time difference could be also attributed to the reduced toxicity of polycationic PS neutralized by polyanionic UFH [2,46]. Nevertheless, PS seems to be the key toxicity mediator, since the effects are similar or even greater when PS is administered without UFH.

The swim bladder of zebrafish larvae shows homology with vertebrate lungs [47]. In our studies, it was deformed under the influence of PS, pointing to potential respiratory toxicity. Normal blood circulation may play an important role in swim bladder development in zebrafish larvae [48,49]. It is possible that PS impaired the development of the swim bladder as a result of heart dysfunction. PS also dose–dependently and strongly influenced the function of the respiratory system in rats. Even at low dose, it increased both respiratory rate and HR and decreased both blood oxygen saturation and the peak exhaled CO_2_, all symptoms characteristic of respiratory failure. Our results and the strong signals from labeled PS observed by others in the lungs shortly after administration, point to this organ as a potential toxicity target of PS [50]. The polycations, such as PS, can damage pulmonary vascular beds via the neutralization of the anionic endothelial surface or by releasing cellular mediators, which increase vascular permeability and disturb the flow of anionic plasma proteins. These observations were confirmed in isolated, perfused rat lung preparations [2,46]. It appears that PS causes charge–dependent toxicity to pulmonary endothelial cells, resulting in both vasoconstriction and increased vascular permeability [2]. The reduction of pulmonary vasoconstriction was attributed to loss of PS cationicity after combination with UFH in perfused rat lung samples [2]. We previously found enlargement of the alveolar sacs with associated hemorrhage in rat lungs one hour after PS administration [51]. The increase of pulmonary vascular resistance can be manifested by pulmonary hypertension. PS and its complexes with UFH produced an increase in pulmonary artery pressure in an isolated cat lung model [52]; the obstruction of the pulmonary vascular bed is one possible explanation [52]. Taking into account the sizes of UFH and PS complexes, one could expect that smaller PS alone will not occlude pulmonary arterioles without the involvement of blood cells. There is no clear information on the size of PS because of its variability and binding with plasma proteins (molecular weights of 5–10 kDa) [53]. We found that PS significantly binds to bovine albumin, and these complexes are quite large, with particle sizes ranging between 1 and 10 µm [54]. It seems that PS can form aggregates with albumin, which could clot the smallest non–muscular pulmonary arterioles measuring 10–25 µm, or at least strongly adhere to the endothelial surfaces of muscular pulmonary microvessels with diameters ranging 25–50 µm. UFH and PS complexes of 0.1 µm are much smaller and cannot clot human arterioles [55]. Although the human pulmonary vessel tree is more branched in comparison to those in rodents, the intravascular volume of capillaries is well conserved, ranging from 85% in human lungs to 86% in mouse lungs [56]. Capillary networks in both species originate from small, non–muscular precapillary arterioles measuring approximately 10–25 µm [57,58]. Potentially, free cationic groups of PS could easily bind to anionic endothelial surfaces of such small vessels, enabling distribution of UFH and PS complexes or PS alone to the most vascularized organs, without the involvement of platelets. Thus, the effects of PS could be different depending on the vessel size. In the bigger vessels, NO released by PS could play a role in the vasodilatation. In the smaller vessels, direct interactions of cationic PS with anionic cell membranes, a mechanism proposed earlier by Chang et al., could mediate acute pulmonary dysfunction [2]. We tried to investigate whether all of these previously reported changes in pulmonary tissue induced by polycations will manifest as general respiratory dysfunction. We found higher respiratory rate, lower blood oxygen saturation, and peak exhaled CO_2_ at the end of the experiment in rats treated with increasing doses of PS. Generally, the effects were stronger when compared to the group treated with UFH and PS. It seems that high concentrations of PS may damage the lungs, or this may occur in the presence of UFH. Our experiment also showed that the symptoms of respiratory failure may be potentiated if the PS/UFH ratio is higher than 1:1. This scenario may take place if PS is administered to patients repeatedly to fully neutralize UFH while UFH is eliminated and its concentration in the blood rapidly decreases.

## 5. Conclusions

We provide clear and consistent results showing the early cardiovascular and respiratory toxicity of PS in zebrafish and rat models. The polycationic nature of PS and the release of NO may be responsible for these disturbances. However, PS administered at low doses in an appropriate ratio with UFH seems to be much safer and should not permanently damage the heart structure and function, as we have shown in long–term observations in mice. PS still remains the only available option when the neutralization of UFH is needed. Appropriate management of PS dosage and more careful monitoring of its potential cardiovascular and respiratory toxicity in patients are necessary, or alternatively a new safer replacement for PS is required.

## Data Availability

Not applicable.
